# Cardiac Arrhythmias and COVID-19: Correlation With Disease Severity

**DOI:** 10.7759/cureus.20507

**Published:** 2021-12-18

**Authors:** Mohammed Mahdi, Vineel Bezawada, Muhammet Ozer, Patrick De Deyne, Bipinpreet Nagra, Bharat Kantharia

**Affiliations:** 1 Internal Medicine, Capital Health Regional Medical Center, Trenton, USA; 2 Clinical Research, Capital Health Regional Medical Center, Trenton, USA; 3 Cardiovascular Disease, Capital Health System, Trenton, USA; 4 Cardiovascular Disease, Mount Sinai Hospital, New York City, USA

**Keywords:** ace-i, oxygen requirement, atrial fibrillation, covid-19, cardiac arrhythmias

## Abstract

Background

Cardiac arrhythmia is one of the life-threatening cardiovascular complications commonly reported in patients hospitalized with coronavirus disease 2019 (COVID-19). We aimed to evaluate the association between cardiac arrhythmias and disease severity based on oxygen requirement.

Methods

In this retrospective observational chart review-based study we recruited 396 patients hospitalized with COVID-19 from March 2020 to May 2020 from two regional medical centers in New Jersey, USA. Patients’ baseline characteristics, secondary diagnoses, and laboratory findings were manually extracted and compared among two groups: patients with cardiac arrhythmias and those without. Poisson regression analysis was used to evaluate the correlation of cardiac arrhythmias and increased oxygen requirement, which are: room air (RA), nasal cannula (NC), high flow nasal cannula (HFNC), and bi-level positive airway pressure ventilation or invasive mechanical ventilation (BIPAP/MV).

Results

The demographic characteristics of the patients were: aged 61 +/- 18.7 years (mean +/- standard deviation); with 56% being male, and 44.9% of African American race. There were 16% patients on RA, 40% on NC, 15% on HFNC, and 29% on BIPAP/MV. The incidence of cardiac arrhythmias was 36.7% (20% pulseless electrical activity (PEA), 13.5% atrial fibrillation (AF). 56% of AF was new-onset arrhythmia. Compared to the RA group, the risk of cardiac arrhythmias was significantly higher in BIPAP/MV (OR 3.3; 95% CI 1.8 - 6.2, p <0.001) and HFNC (OR 2.9; 95% CI 1.5-5.7, p0.001), but not in NC group (OR 0.95; 95% CI 0.4-1.8, p0.89).

Compared to patients without arrhythmias, patients with arrhythmias were older (mean age 71 vs. 56 years, p <0.001) and had more comorbidities (Charlson comorbidity index (CCI), 4.7 vs. 2.9, p <0.001). The continued therapy of angiotensin-converting enzyme inhibitors or angiotensin-II receptor blockers did not seem to be associated with increased or decreased risk of cardiac arrhythmias.

Conclusion

The incidence of cardiac arrhythmias among hospitalized COVID-19 patients was 36.7% with PEA being common in patients who succumbed to death, and AF in those patients who survived. The incidence of cardiac arrhythmias positively correlated with disease severity based on oxygen requirement and was higher among patients requiring HFNC or BIPAP/MV.

## Introduction

The coronavirus disease 2019 (COVID-19) pandemic due to widespread infection with novel severe acute respiratory syndrome coronavirus-2 (SARS-CoV-2) has led to significant overall mortality and morbidity globally [1-2]. In the United States (US) alone, the total number of COVID-19 cases reached 35,129,562, with total US deaths of 613,670 as of August 2021 [3].

Cardiovascular complications were commonly reported in hospitalized patients with COVID-19, initially reported by Huang et al. [4]. Subsequently, several similar observational studies were conducted to document this co-incidental finding. Recently, a meta-analysis study was conducted by Kunutsor et al. analyzing the data from 5815 patients diagnosed with COVID-19 using 17 observational studies and found that the cardiac arrhythmias pooled incidence was 9.3% [5].

US-based clinical studies found the incidence of cardiac arrhythmias to be approximately 14% [6-7]. In an international study conducted in 3011 patients from 13 countries, the incidence of cardiac arrhythmias was lower at 8.6%, with atrial fibrillation (AF) being the most common (4.7%) [8].

In our study, we aimed to (i) describe the incidence of cardiac arrhythmias, (ii) study the association with disease severity based on oxygen requirement, and (iii) describe the clinical characteristics of the patients with cardiac arrhythmias among hospitalized patients with COVID-19 from two regional medical centers in New Jersey, USA.

## Materials and methods

Study design and populations

We used a retrospective observational chart review-based study design that included patients older than 18 years of age and were hospitalized between March 23 and May 6, 2020, with a diagnosis of COVID-19 confirmed by SARS-CoV-2 polymerase chain reaction (PCR) testing of the nasopharyngeal swab. The patients who were not on cardiac telemetry were excluded. The patient population was two regional medical centers in New Jersey, USA. 

Ethics

The study was conducted according to the guidelines of the Declaration of Helsinki and approved by the Institutional Review Board of Capital Health System, approval number CH830. 

Data collection

We collected demographic data from the electronic medical record (EMR), including race, ethnicity, age, gender, insurance status. Charlson comorbidity index (CCI) was used to document comorbidities, including a history of myocardial infarction (MI), congestive heart failure (CHF), peripheral vascular disease, cerebral vascular accident (CVA) or transient ischemic attack, dementia, chronic obstructive pulmonary disease, connective tissue disease, peptic ulcer disease, liver disease, diabetes mellitus, hemiplegia, moderate to severe chronic kidney disease (CKD), solid tumor, leukemia, lymphoma, or acquired immunodeficiency syndrome. 

Progress notes and discharge summaries were reviewed for concomitant acute illnesses, specifically pulmonary embolism/deep venous thrombosis, superimposed bacterial pneumonia, acute coronary syndrome (acute ST or non-ST elevation myocardial infarction), acute kidney injury (AKI), and acute cerebrovascular accident.

The laboratory values were reviewed at the time of presentation, including ferritin, C-reactive protein, fibrinogen, D-dimer, procalcitonin, lactate dehydrogenase, N-terminal pro-brain natriuretic peptide (NT-proBNP), troponin-I, creatine kinase, lactic acid, white blood cells count, lymphocyte percentage, neutrophil percentage, sodium, aspartate transaminase, and alanine transaminase. Also, the lowest level of magnesium and potassium during hospitalization were entered. A significantly prolonged QTc as the duration of more than 500 msec from the ECG of presentation day was documented as well; we verified the therapy received, such as remdesivir, convalescent plasma, tocilizumab, dexamethasone, methylprednisolone, hydroxychloroquine, azithromycin, paralytics, and vasopressors. 

Outcomes

We used information gathered from EMR and the daily telemetry logs for each patient to verify the presence or absence of AF, atrial flutter, supraventricular tachycardia, non-sustained ventricular tachycardia, sustained ventricular tachycardia, first degree atrioventricular (AV) block, second degree AV block, third-degree AV block, clinically significant bradycardia, ventricular fibrillation, and pulseless electrical activity/asystole cardiac arrest. The presence of one or more defined the presence of an arrhythmia. 

The highest level of oxygen support received by the patient during the hospital stay was noted by reviewing the nursing charts and respiratory therapist notes. We divided the patients into four groups: patients receiving room air (RA) (no oxygen support), a nasal cannula (NC) (1-6 liters/min oxygen), high flow nasal cannula (HFNC) (>6 liters/min oxygen), or bilevel positive airway pressure/invasive mechanical ventilation (BIPAP/MV).

Statistical analysis

The data analysis was performed with IBM SPSS Statistics for Windows, Version 26.0 (Released 2019, IBM Corp., Armonk, New York). We included the demographic and clinical data, and when data points were not obtained, they were recorded as missing. We created frequency tables for the nominal and ordinal variables and descriptive statistics to calculate the means and variance for continuous variables. Statistical relationships and comparisons between the arrhythmia group and no arrhythmia groups were performed using Chi-Square tests for nominal and ordinal variables. A two-way Student’s t-test was used for continuous variables; for each series of analyses, the p-value was set at 0.05 to determine significant differences. To predict the increased risk of cardiac arrhythmias (as dependent variable) with increased oxygen requirements (relative to RA), we used an unadjusted single Poisson regression analysis, which provided us with the relative risk (including confidence intervals and significance). Continuous variables are presented as means and standard deviations (SD) and categorical variables as counts and percentages.

## Results

A total of 396 patients were included in the study. The mean age was 61.8 years (±18.7), and 223 patients (56%) were male. 178 (45%) were African Americans, and 109 (27.5%) were Caucasians. The majority (86.5%) of our patients were insured (private insurance, Medicare, or Medicaid), with only 53 patients (13.5%) classified as non-insured. The most common comorbidity was diabetes mellitus (n=148, 37.5%). Superimposed bacterial pneumonia and acute kidney injury represented the most common acute secondary diagnoses (n=133, 33.7%, and n=130, 32.9%, respectively). The received targeted therapy was hydroxychloroquine (n= 171, 43.3%) and azithromycin (n= 169, 42.8%). Corticosteroids were used mostly in the form of intravenous methylprednisolone (n= 115, 29.1%). Few patients received remdesivir (n=14, 3.5%), convalescent plasma (n= 26, 6.6%), and tocilizumab (n=17, 4.3%). According to their oxygen requirement, patients on NC up to 6 L/min were the largest group (n=156, 39.8%) patients followed by BIPAP/MV group (114, 29.1%), RA group (64, 16.3%), and HFNC group (58, 14.8%). The mortality rate was 27.3%, and the average total length of stay was 12.6 ± 13.2 days (Table 1).

**Table 1:** Baseline characteristics of patients with COVID-19 BIPAP: bi-level positive airway pressure ventilation, MV: mechanical ventilation

Variable	Value
Age – year ± SD	61.8 ± 18.7
Male sex – no. (%)	223 (56.3)
Race – no. (%)	
African American	178 (44.9)
Caucasian	109 (27.5)
Hispanic	41 (10.4)
Asian	15 (3.8)
No medical insurance – no. (%)	53 (13.5)
BMI – mean kg/m^2^ ± SD	29.6 ± 10.7
Comorbidities – no. (%)	
History of myocardial infarction	32 (8.1)
Congestive heart failure	45 (11.4)
Peripheral vascular disease	21 (5.3)
History of Cerebrovascular accident	54 (13.7)
Dementia	72 (18.2)
Chronic obstructive pulmonary disease	45 (11.4)
Connective tissue disease	5 (1.3)
Peptic ulcer disease	4 (1)
Liver disease	
Mild	14 (3.5)
Moderate - severe	1 (0.3)
Diabetes Mellitus	
Uncomplicated	127 (32.2)
With end-organ damage	21 (5.3)
Hemiplegia	9 (2.3)
Chronic kidney disease	65 (16.4)
History of solid tumor	26 (6.6)
History of leukemia	2 (0.5)
History of lymphoma	2 (0.5)
Acquired immunodeficiency syndrome	1 (0.3)
History of atrial fibrillation	38 (9.7)
Acute secondary diagnosis – no. (%)	
Venous thromboembolism	11 (2.8)
Superimposed bacterial pneumonia	133 (33.7)
Acute coronary syndrome	9 (2.3)
Cerebrovascular accident	5 (1.3)
Acute kidney injury	130 (32.9)
Oxygen requirement – no. (%)	
Room air	64 (16.3)
Nasal canula	156 (39.8)
High flow nasal canula	58 (14.8)
BIPAP/MV	114 (29.1)
Disposition – no. (%)	
Discharged to home	171 (43.2)
Discharged to facility	113 (28.5)
Expired	108 (27.3)
Left against medical advice	4 (1)
Total Length of stay – days ± SD	12.6 ± 13.2
Targeted therapy – no. (%)	
Remdesivir	14 (3.5)
Convalescent plasma	26 (6.6)
Tocilizumab	17 (4.3)
Dexamethasone	22 (5.6)
Methylprednisolone	115 (29.1)
Hydroxychloroquine	171 (43.3)
Azithromycin	169 (42.8)
Vasopressors	78 (19.7)
QTc >500 msec – no. (%)	42 (11.1)

**Figure 1:**
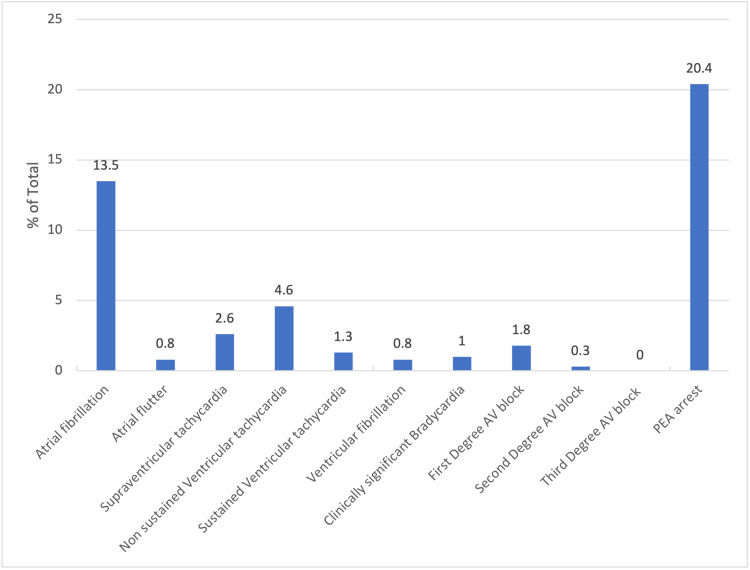
Cardiac arrhythmia types and rates AV: atrioventricular, PEA: pulseless electrical activity

Cardiac arrhythmias occurred in 144 patients (36.7%). PEA was observed as fatal arrhythmia in 80 patients (20%). AF was the most common non-arrest cardiac arrhythmia in 53 patients (13.5%), of which new-onset AF occurred in 30 patients (7.6%). Less common arrhythmias were non-sustained ventricular tachycardia occurred in 18 (4.6%) and supraventricular tachycardia in 10 (2.6%) (Figure 1).

Compared to patients who survived and had no cardiac arrhythmias, those who developed cardiac arrhythmias and those who sustained cardiac arrest were older (mean age 71.8 ± 14 vs. 58.9 ± 18, p <0.001) and White/Caucasian (40.8% vs. 20.6%, p <0.001). Although higher in rate among males, the variation was statistically insignificant (male:female ratio 1.6:1 vs. 1.1:1; p 0.09). The CCI was higher among patients with cardiac arrhythmias (4.7 ± 2.5 vs. 2.9 ± 2.6, p <0.001). Patients with cardiac arrhythmias were more likely to have a history of MI, CHF, peripheral vascular disease, CVA, dementia, diabetes mellitus, and CKD with statistical significance (Table 2). Patients with cardiac arrhythmias were also more likely to have a concomitant acute illness, mainly superimposed bacterial pneumonia (47.2% vs. 26.3%, p <0.001) and acute kidney injury (54.2% vs. 20.6%, p <0.001).

**Table 2:** Baseline differences between patients with and without cardiac arrhythmias

Variable	Arrhythmia	No arrhythmia	P value
Age – year ± SD	71.8 ± 14.2	58.9 ± 18.9	<0.001
Gender			0.091
Male – no. (%)	90 (62.5%)	132 (53.2%)	
Female – no. (%)	54 (37.5%)	116 (46.8%)	
Race			<0.001
Caucasian – no. (%)	58 (40.8%)	51 (20.6%)	
Hispanic – no. (%)	7 (4.9%)	33 (13.4%)	
African American – no. (%)	63 (44.4%)	113 (45.7%)	
Asian – no. (%)	4 (2.8%)	10 (4%)	
Other – no. (%)	10 (7%)	40 (16.2%)	
Comorbidities			
Charlson Comorbidity Index – point ± SD	4.7 ± 2.5	2.9 ± 2.6	<0.001
History of myocardial infarction – no. (%)	21 (14.6%)	11 (4.5%)	0.001
Congestive heart failure – no. (%)	26 (18.1%)	19 (7.7%)	0.003
Peripheral vascular disease – no. (%)	13 (9%)	8 (3.2%)	0.019
History of Cerebrovascular accident – no. (%)	29 (20.1%)	23 (9.3%)	0.003
Dementia – no. (%)	42 (29.2%)	29 (11.7%)	<0.001
Chronic obstructive pulmonary disease – no. (%)	22 (15.3%)	23 (9.3%)	0.099
Liver disease, mild – no. (%)	8 (5.6%)	6 (2.4%)	0.208
Diabetes Mellitus – no. (%)	73 (51.1 %)	75 (30.4%)	<0.001
Chronic kidney disease – no. (%)	39 (27.1)	26 (10.5%)	<0.001
Solid tumor – no. (%)	14 (9.7%)	12 (4.9%)	0.09
Body mass index – no. (%)	28.1 ± 8.5	30.5 ± 11.8	0.021
Concomitant Acute illness			
Venous Thromboembolism – no. (%)	1 (0.7%)	10 (4%)	0.061
Superimposed bacterial pneumonia – no. (%)	68 (47.2%)	65 (26.3%)	<0.001
Acute coronary syndrome – no. (%)	4 (2.8%)	5 (2%)	0.73
Cerebrovascular accident – no. (%)	2 (1.4 %)	3 (1.3%)	1.000
Acute kidney injury – no. (%)	78 (54.2%)	51 (20.6%)	<0.001
QTc > 500 msec – no. (%)	22 (15.7%)	20 (8.5%)	0.041
Lavoratory values			
Ferritin – SI ± SD	2487 ± 7497	2174 ± 4537	0.607
C-reactive protein – SI ± SD	17.2 ± 13	13.6 ± 11	0.007
Fibrinogen – SI ± SD	641.4 ± 214	660 ± 217	0.474
D-dimer – SI ± SD	5.1 ± 6.0	3.2 ± 5.1	0.003
Procalcitonin – SI ± SD	3.31 ± 16.5	11.3 ± 119	0.434
Lactate dehydrogenase – SI ± SD	956 ± 4544	468 ± 347	0.111
NT-pro brain natriuretic peptide – SI ± SD	9719 ± 31664	2352 ± 8495	0.055
Troponin-I – SI ± SD	3.03 ± 21.86	0.11 ± 0.58	0.196
lactic acid – SI ± SD	2.3 ± 2.5	1.8 ± 2.3	0.065
White blood cell count – SI ± SD	9.4 ± 6.0	8.0 ± 4.4	0.01
Aspartate Aminotransferase – SI ± SD	416 ± 2544	88 ± 223	0.049
Alanine Aminotransferase – SI ± SD	168 ± 894	66 ± 123	0.084
Creatine kinase – SI ± SD	4262 ± 23067	682 ± 1597	0.218
Potassium – SI ± SD	3.6 ± 0.7	3.5 ± 0.4	0.219
Magnesium – SI ± SD	2.0 ± 0.4	2.0 ± 0.3	0.596
Treatment			
Remdesivir – no. (%)	9 (6.3%)	5 (2%)	0.045
Convalescent plasma – no. (%)	10 (6.9%)	16 (6.5%)	0.837
Methylprednisolone – no. (%)	53 (36.8%)	62 (25.1%)	0.016
Hydroxychloroquine – no. (%)	69 (47.9%)	101 (40.9%)	0.204
Azithromycin – no. (%)	61 (42.4%)	107 (43.3%)	0.916
Tocilizumab – no. (%)	5 (3.5%)	12 (4.9%)	0.614
Paralytics – no. (%)	21 (14.6%)	12 (4.9%)	0.001
Vasopressors – no. (%)	51 (35.4%)	27 (10.9%)	<0.001
Angiotensin-converting enzyme inhibitor or angiotensin receptor blocker use – no. (%)	20 (14%)	33 (13.3%)	0.706
Oxygen Requirement			<0.001
Room air – no. (%)	12 (8.3%)	52 (21%)	
Nasal cannula up to 6 L/min – no. (%)	28 (19.4%)	128 (51.6%)	
High Flow Nasal cannula up to 60L/min – no. (%)	32 (22.2%)	26 (10.5%)	
Bilevel Positive Airway Pressure or mechanical ventilation – no. (%)	72 (50%)	42 (16.9%)	
Total Length of stay – days	19.1	10.7	<0.001

A significantly prolonged QTc (duration equal to or > 500 msec) captured from a 12 leads electrocardiogram at presentation was more prevalent in patients with cardiac arrhythmias (15.7% vs. 8.5%, p 0.02). Among inflammatory and cardiac biomarkers, only C-reactive protein, D-dimer, and aspartate aminotransferase differed significantly between the two groups, with being more elevated among the arrhythmia group (Table 2). Patients with cardiac arrhythmias were more likely to have received remdesivir, methylprednisolone, paralytics, and vasopressors. Between groups, differences were not seen regarding convalescent plasma, hydroxychloroquine, azithromycin, and tocilizumab. The chronic use of either an angiotensin-converting enzyme (ACE) inhibitor or an angiotensin-II receptor blocker (ARB) was not associated with the risk of developing, or protecting against, cardiac arrhythmias. 

**Figure 2:**
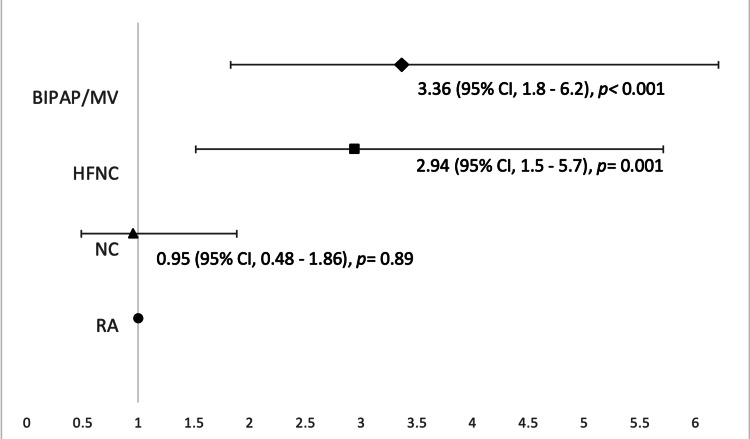
Relative risks of cardiac arrhythmias among patients with different oxygen supplement settings BIPAP: bi-level positive airway pressure ventilation, HFNC: high flow nasal cannula, MV: mechanical ventilation, NC: nasal cannula, RA: room air

Cardiac arrhythmias were more likely to occur in patients who were on BIPAP/MV (n=72, 50%) than patients on RA, NC, or HFNC (8.3%, 19.4%, 22.2%, respectively; p <0.001). The risk of cardiac arrhythmias was significantly higher among HFNC (Relative risk (RR) 2.9, 95% CI 1.5 - 5.7; p <0.001) and BIPAP/MV (RR 3.1, CI 1.8 - 6.2; p <0.001) groups compared to the RA group. This association was not seen in NC group (RR 0.95, CI 0.48 - 1.88; p=0.89) (Figure 2).

## Discussion

Our retrospective observational study of 396 patients hospitalized with COVID-19 indicated that 36% of the patients developed cardiac arrhythmias, most commonly PEA cardiac arrest (20%), mostly upon withdrawal of care after the change of code status (9.4%). The second most common cardiac arrhythmia was AF (13.5%), of which 7.6% was new-onset. Recently, Musikantow et al. reported that AF or atrial flutter occurs in the incidence of approximately 13%, of which 4% was new-onset atrial arrhythmias [9]. The incidence of atrial arrhythmia, specifically new-onset, in our patients’ population seems higher than what is reported in the literature, perhaps due to more complications and comorbidities.

The potential causes for cardiac arrhythmias in patients with COVID-19 could be acute coronary syndrome, myocarditis, stress-induced cardiomyopathy, systemic inflammation/cytokine storm syndrome, and/or hypoxia associated with severe disease [9-10]. Our data indicate that the incidence of cardiac arrhythmias was positively associated with disease severity based on oxygen requirement. The incidence of arrhythmias increases from 8.3% in the RA group to 19.4%, 22.2%, and 50% in NC, HFNC, and BIPAP/MV groups, respectively. This risk is considered higher than cardiac arrhythmias in hospitalized patients with community-acquired pneumonia, which is 4.7% reported in a meta-analysis study [11]. The risk of cardiac arrhythmias was significantly higher among patients who received HFNC (OR 2.9, CI 1.5 - 5.7; p 0.000) or BIPAP/MV (OR 3.1, CI 1.8 - 6.2; p 0.000), but was not observed in the NC group (OR 0.95, CI 0.48 - 1.88; p 0.89) when compared to RA reference group. This relation suggests that cardiac arrhythmias are related to the level of oxygen requirement, which is a proxy for the disease severity. 

In our patients, the arrhythmia group was more likely to have received remdesivir, methylprednisolone, paralytics, and vasopressors. The patients in the arrhythmia group were more severely affected by COVID-19, requiring vasopressor supportive therapy, which is a potential cause for arrhythmia. Since steroids and remdesivir were primarily used for severely affected patients, the arrhythmia could be related to the disease severity. 

Our study findings are similar to that of a study conducted by Bhatla et al. with 700 patients, which concluded that cardiac arrest and arrhythmias are more likely a consequence of systemic inflammation than direct cardiac injury by a coronavirus, given that most of the events occurred in patients receiving care in the ICU and almost no events in the non-ICU level of care [11]. While in another observational study from 50 patients hospitalized for COVID-19, Angeli et al. concluded that the development of new ECG abnormalities was irrelevant to the disease severity [12]. Recently, cardiac magnetic resonance imaging (CMRI) findings were consistent with myocarditis and reported in 60% and 57% of patients recovered from COVID-19 in two small studies [13-14]. Thus, subclinical myocarditis is a potential complication of COVID-19. However, additional specialized studies like echocardiography, CMRI, and cardiac angiography were mainly deferred because of strict isolation precaution measures. 

In our study, the patients in the cardiac arrhythmia group were older and had more comorbidities with a higher CCI; among those comorbidities, diabetes mellitus, CHF, history of MI, history of CVA, peripheral vascular disease, CKD, and dementia were reported more often among the cardiac arrhythmia group. Patients with AKI and superimposed bacterial pneumonia were more likely to develop cardiac arrhythmias. These comorbidities could have contributed to the risk of systemic inflammation and eventually metabolic stress on the cardiomyocyte and the cardiac conduction system. From the biomarkers, C-reactive protein and d-dimer were more elevated among the cardiac arrhythmia group. Altogether, these findings make systemic inflammation a favorable potential mechanism of cardiac arrhythmias.

Some of the medications used in the treatment of COVID-19 are known to cause QTc prolongation and thus a risk of cardiac arrhythmias, specifically Torsades de pointes [15]. In our patient population, while QTc prolongation was more often reported in the cardiac arrhythmia group (15.7% vs. 8.5%, p 0.041), the use of hydroxychloroquine and azithromycin did not differ significantly between the two groups of our patients. Although QTc prolongation is a known effect of hydroxychloroquine therapy, taken alone or with concomitant azithromycin, in hospitalized patients with COVID-19 [16], the risk of ventricular tachyarrhythmias did not differ significantly between hydroxychloroquine and placebo in a randomized controlled trial of 479 patients [17]. 

Electrolyte disturbances, especially hypomagnesemia and hypokalemia, are essential in setting the threshold low for cardiac arrhythmias [18]. In our patients, potassium and magnesium levels during the hospitalization did not differ significantly between the two groups and were around the normal level.

In our study, there was no significant difference between the two groups regarding angiotensin-converting-enzyme inhibitors (ACEI) or angiotensin receptor blockers (ARB) use. There are two theories in lieu of resuming renin-angiotensin system (RAS) inhibitory medications, ACEI or ARB. The first suggests that RAS inhibitors cause feedback increase in angiotensin-converting enzyme-2 (ACE2) receptors in the pulmonary system, which theoretically leads to an enhanced viral entry and thus more severe disease [19-20]. On the other hand, the second theory states that SARS-CoV interaction with ACE2 receptors will lead to exhaustion, leaving the ACE and angiotensin levels high enough to result in multi-system inflammation, and therefore, ACEI or ARB use is associated with less inflammation and thus less severe disease from COVID-19 [21]. A large study on almost 825,000 patients carried by Jaejin et al. concluded that neither ACEI nor ARB use was associated with an increased likelihood of COVID-19 infection [22].

Limitations

Our study is a retrospective cohort design in which the data were collected via electronic medical records review and manual abstraction; we used the “Covid-19 ???” diagnostic code to collect the patient data irrespective of the presence of cardiac or respiratory events, hence minimizing any bias. With respect to the PEA cardiac arrest incidence, this was mainly due to the withdrawal of care that raised the incidence of any cardiac arrhythmia in general. Laboratory values and QTc interval were obtained from the day of admission, which could potentially underestimate significant findings as to the disease progress and multi-organ failure. Finally, detailed studies that contribute to the final diagnosis, including echocardiogram, cardiac angiography, and cardiac MRI, were not performed due to the strict isolation precautions. 

## Conclusions

The incidence of cardiac arrhythmias among hospitalized COVID-19 patients increases with the disease severity when associated with HFNC or BIPAP/MV. The most common cardiac arrhythmia other than PEA is AF. Likely multiple comorbidities and systemic inflammation play a significant role in cardiac arrhythmias incidence.
